# Integrated network pharmacology and hepatic metabolomics to reveal the mechanism of *Acanthopanax senticosus* against major depressive disorder

**DOI:** 10.3389/fcell.2022.900637

**Published:** 2022-08-05

**Authors:** Xinyi Gu, Guanying Zhang, Qixue Wang, Jing Song, Ying Li, Chenyi Xia, Ting Zhang, Li Yang, Jijia Sun, Mingmei Zhou

**Affiliations:** ^1^ Institute for Interdisciplinary Medicine Sciences, Shanghai University of Traditional Chinese Medicine, Shanghai, China; ^2^ Shanghai Frontiers Science Center of TCM Chemical Biology, Institute of Interdisciplinary Integrative Medicine Research, Shanghai University of Traditional Chinese Medicine, Shanghai, China; ^3^ School of Pharmacy, Shanghai University of Traditional Chinese Medicine, Shanghai, China; ^4^ Department of Physiology, School of Basic Medical Sciences, Shanghai University of Traditional Chinese Medicine, Shanghai, China; ^5^ Department of Mathematics and Physics, School of Pharmacy, Shanghai University of Traditional Chinese Medicine, Shanghai, China

**Keywords:** *Acanthopanax senticosus* Harms, major depressive disorder, metabolomics, network pharmacology, molecule docking

## Abstract

**Objective:** *Acanthopanax senticosus* (Rupr. et Maxim.) Harms (ASH) is a traditional herbal medicine widely known for its antifatigue and antistress effects, as well as tonifying qi, invigorating spleen and kidney, and tranquilizing the mind. Recent evidence suggests that ASH has a therapeutic effect on major depressive disorder (MDD), but its mechanism is still unclear. The current study aimed to investigate the effect of ASH on MDD and potential therapeutic mechanisms.

**Materials and Methods:** The chemical compound potential target network was predicted based on network pharmacology. Simultaneously, chronic unpredictable mild stress (CUMS) model mice were orally administrated ASH with three dosages (400, 200, and 100 mg/kg) for 6 weeks, and hepatic metabolomics based on gas chromatography–mass spectrometry (GC–MS) was carried out to identify differential metabolites and related metabolic pathways. Next, the integrated analysis of metabolomics and network pharmacology was applied to find the key target. Finally, molecular docking technology was employed to define the combination of the key target and the corresponding compounds.

**Results:** A total of 13 metabolites and four related metabolic pathways were found in metabolomics analysis. From the combined analysis of network pharmacology and metabolomics, six targets (DAO, MAOA, MAOB, GAA, HK1, and PYGM) are the overlapping targets and two metabolic pathways (glycine, serine, and threonine metabolism and starch and sucrose metabolism) are the most related pathways. Finally, DAO, MAOA, MAOB, GAA, HK1, and PYGM were verified bounding well to their corresponding compounds including isofraxidin, eleutheroside B1, eleutheroside C, quercetin, kaempferol, and acacetin.

**Conclusion:** Based on these results, it was implied that the potential mechanism of ASH on MDD was related to the regulation of metabolism of several excitatory amino acids and carbohydrates, as well as the expression of DAO, MAOA, MAOB, GAA, HK1, and PYGM.

## 1 Introduction

Major depressive disorder (MDD) is a complicated and severe psychological disorder, characterized by low mood, reduced activity, and cognitive dysfunction. More than 350 million people worldwide suffer from depression (Paulina et al., 2018). According to the World Health Organization report, nearly 800,000 people die by suicide every year (WHO, 2020). Depression reduces the quality of life and has become a health burden for families and society (Guo et al., 2017).

Even though the monoamine transmitter hypothesis is widely accepted, the pathological mechanism of depression is still uncertain. In addition, the neuroendocrine hypothesis, abnormal feedback regulation of the hypothalamus–pituitary–adrenal (HPA) axis, and social stress are also related to depression (Cai et al., 2015; Afridi and Suk, 2021). Based on the research of these disease mechanisms, various antidepressant drugs have been developed, including selective serotonin reuptake inhibitors, tricyclic antidepressants, serotonin–norepinephrine reuptake inhibitors, monoamine oxidase inhibitors, and atypical antidepressants such as benzodiazepines (Strawn et al., 2018). Although more than a dozen of antidepressants are available, most individuals with depression have no response to these treatments.

Traditional Chinese medicines, with the characteristics of multiple effects, multiple compounds, and multiple targets, have been widely used in the treatment of depression (Gu et al., 2021). *Acanthopanax senticosus* (Rupr. et Maxim.) Harms (ASH), also known as “Siberian ginseng,” is a kind of hardy shrub that originates in China, Korea, Russia, and Japan, famous for its antifatigue and antistress effect. As a traditional Chinese herbal medicine, it is widely known for tonifying Qi. Qi is the vital energy of life, and Qi deficiency is mostly caused by over work, improper diet, aging, frailty, and chronic illness, and generally manifests as physical weakness, pale complexion, shortness of breath, limb weakness, dizziness, insomnia, sweating, and low voice. The manifestation of Qi deficiency is similar to physical weakness in modern medicine, accompanied by low-energy metabolic levels and pre-depression symptoms. The indication of ASH in traditional Chinese medicine is physical weaknesses, fatigue, loss of appetite, and insomnia. Now, ASH is commonly used in heart disease, hypertension, allergies, diabetes, rheumatoid arthritis, and neurodegenerative diseases (Yi et al., 2002; Liu et al., 2008; Takahashi et al., 2014; Liu et al., 2018). In addition, ASH is also widely used in the treatment of depression. Shugan Jieyu Capsule, composed of ASH and *Hypericum perforatum*, is one of the Chinese patent medicines for treating depression in China (Zhang X. et al., 2014). The mixture of chlorogenic acid and (+)-syringaresinol-di-O-β-D-glucoside, ingredients in ASH, could induce anxiolytic behavior and regulate the autonomic nervous system (Miyazaki et al., 2020). Qi et al. (2020) reported ASH extract’s antidepressant effect by improving the contents of dopamine (DA), norepinephrine (NE), and 5-hydroxytryptamine (5-HT). Moreover, Bhaktaprasad and Dongwook (2014) showed that ASH extract restored both altered c-fos expression and HPA activity, which have beneficial effects on depression behaviors. The anti-inflammatory, antistress, and neuroprotective effects of ASH are also very beneficial in treating depression (Kimura and Sumiyoshi 2004; Zhang S. et al., 2014; Han et al., 2016).

Neurological diseases, including MDD, are closely related to metabolic disorders (Bhadra et al., 2021). Chronic unpredictable mild stress (CUMS) is one of the most commonly used preclinical models for understanding the onset and progression of MDD (Willner, 2017). CUMS can well induce several features of human pathology, such as altered circadian rhythms, anhedonia, and increased anxiety and hopelessness (Bosch et al., 2022). Behavioral tests such as sucrose preference test (SPT), tail suspension test (TST), forced swimming test (FST), and open-field test (OFT) are commonly used to assess the depression phenotype. Metabolomics has provided new insights into pathogenic mechanisms, treatment responses, and biomarker verification related to MDD (Duan and Xie, 2020; Gu et al., 2021). Although in general, the brain is a first priority organ for depression studies, traditional Chinese medicine theory believes that one of the most important pathogeneses of depression is “the stagnation of Liver-Qi” (Chen et al., 2020). The liver is the hub of metabolism and energy substrate homeostasis. Studies have proved that mental illness (including depression) is associated with liver disorders (Russ et al., 2015; Wu et al., 2016). Disturbances in the liver may cause HPA axis dysfunctions, hippocampal neurogenesis impairments, and neuroplasticity and neurosteroid synthesis alterations. These pathophysiological processes may have influence on related brain regions, and exacerbate symptoms of depression (Kimoto et al., 2001; Leonard, 2001; Barger et al., 2005; Koo et al., 2010). Based on these theories, many metabolomics studies on CUMS-induced depression have used liver tissue as the research object (Chen et al., 2015; Jia et al., 2016; Jia et al., 2017; Liu et al., 2021).

Network pharmacology, a newly developed technology of systems biology, connects the mechanism of chemical compounds, drug targets and diseases, as well as visualizes, systematizes, and informs the process principles of complex disease treatment (Zhang et al., 2016). From the perspective of systems and networks, network pharmacology has been widely used in studying the relevance between herbs and diseases (Liu et al., 2019). Although pharmacological and clinical studies have shown that ASH has a relatively definite antidepressant effect such as restoring altered c-*fos* expression and HPA activity (Gaire and Lim 2014) or modulating the central monoaminergic neurotransmitter system and CREB protein expression (Jin et al., 2013), the chemical basis and mechanism are still unclear. Network pharmacology is expected to provide new ideas.

**FIGURE 1:**
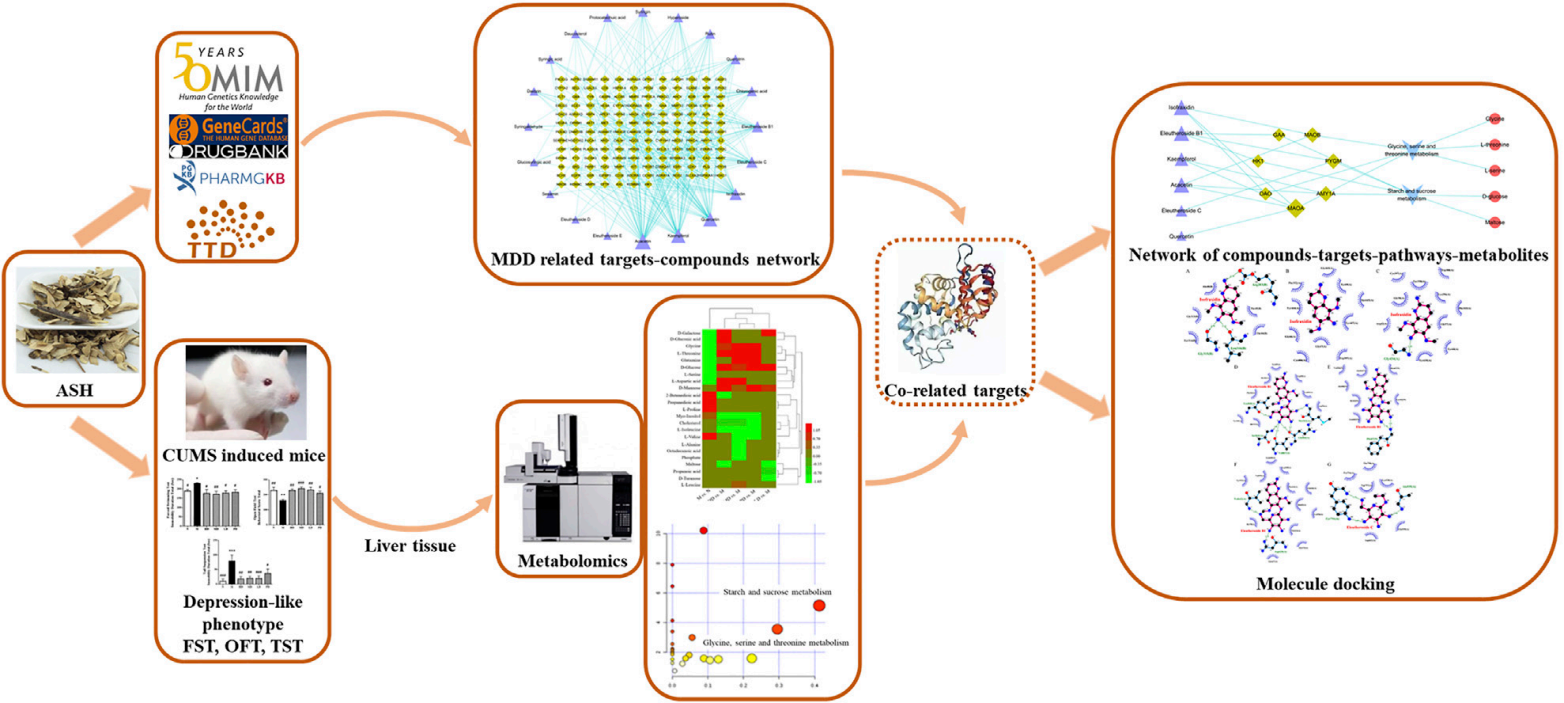
Work scheme of this study. ASH: *Acanthopanax senticosus* (Rupr. et Maxim.) Harms; FST: forced swimming test; MDD, major depressive disorder; OFT, open-field test; TST, tail suspension test.

At present, there are still few metabolomic studies and network pharmacology technology target prediction of ASH treating depression. In this study, hepatic metabolomics based on gas chromatography–mass spectrometry (GC–MS) combined with network pharmacology was first applied to find biomarkers, related pathways, hub targets, and key compounds of ASH on MDD. Then, a molecular docking method was used to identify the binding affinity of each compound and target, preliminary exploring the mechanism of ASH on MDD. The flowchart of this study design is presented in Figure 1.

## 2 Methods and materials

### 2.1 Network pharmacology

#### 2.1.1 Screening of chemical compounds and targets of ASH

The major chemical constituents of ASH were determined by the literature on ASH chemical composition study. The SwissTargetPrediction database (http://www.swisstargetprediction.ch/) was used to obtain potential targets for each chemical compound. The UniProt database (https://www.UniProt.org/UniProt/) was employed to define each abbreviation of the proteins of ASH. The information on ADME including oral bioavailability (OB), drug-likeness (DL), gastrointestinal (GI) absorption, and blood–brain barrier (BBB), was obtained from the SwissADME database (http://www.swissadme.ch/) and TCMSP database (http://lsp.nwu.edu.cn/tcmsp.php).

#### 2.1.2 MDD-associated target prediction

“Major depressive disorder” was imported to the database of OMIM (https://www.OMIM.org/), GeneCard (https://www.genecards.org/), DrugBank (https://www.DrugBank.ca), PharmGkb (https://www.PharmGkb.org/), and Therapeutic Target database (http://bidd.nus.edu.sg/bidd-databases/TTD/TTD.asp). Combined the targets searched from each database and deleted duplicate ones, the rest were targets for MDD. The shared targets for both the active compounds in ASH and the disease were selected as the possible targets of ASH in treating MDD. The Venny 2.1 (http://bioinfogp.cnb.csic.es/tools/venny/index.html) was used to map the targets between ASH and MDD.

#### 2.1.3 Construction of network

MDD targets and compounds in ASH, which have been screened, were introduced into Cytoscape software to build a visual network.

### 2.2 Metabolomics

#### 2.2.1 Experimental reagents and equipment

Methanol (Sigma; Lot # WXBC2211V), chloroform (Sinopharm Chemical Reagent Co., Ltd.; batch number 20161128), pyridine (Sinopharm Chemical Reagent Co., Ltd.; batch number 20140424), heptadecanoic acid (Aladdin; Lot number: K1325026), O-methoxyamine-HCl (SUPELCO; Lot # LB66506). N, O-Bis (trimethylsilyl) trifluoro-acetamide (BSTFA with 1% TMCS) (Sigma-Aldrich; Lot # BCBWA670). Tabellae Acanthopanacis Senticosi was purchased from Shan Xi Yun Peng Pharmaceutical Co. Ltd., and fluoxetine hydrochloride dispersible tablets were purchased from Eli Lilly and Company. The quality control of ASH was carried out by high-performance liquid chromatography (HPLC), according to the method of Zhu et al. (2011). The level of characteristic chemicals for eleutheroside E and syringin was 2.038 and 3.357 mg/g, respectively (Supplementary Figure S1).

GC–MS (6890N-5975B; Agilent; United States), automatic sample rapid grinder (TissueLyser-24; Shanghai Jingxin Industrial Development Co., Ltd.), vortex mixer (Vortex-Genie 2; Scientific Industries; United States), high-speed refrigerated centrifuge (Centrifuge 5415R; Eppendorf; Germany), nitrogen blowing apparatus (SBH130D/3; Stuart; United Kingdom), oscillating low-temperature incubator (Enviro-Genie; Scientific Industries; United States).

Methanol–water–chloroform: prepared in a ratio of 5∶2∶2 (v/v/v); heptadecanoic acid methanol solution (1.0 mg/ml): 10 mg of heptadecanoic acid dissolved in 10 ml of methanol; methoxyamine pyridine hydrochloride solution (15 mg/ml): 150 mg of O-methoxyamine–HCl dissolved in 10 ml of pyridine.

#### 2.2.2 Animals

A total of 36 male ICR mice (20–22 g, Shanghai, China, approval number: 2014-0008) were provided by the Laboratory Animal Center of Shanghai University of Traditional Chinese Medicine. All mice were free to food (AIN-93 purified standard diets) and water under the barrier system and bred adaptively for 7 days. Animal welfare is strictly implemented in accordance with the “The Guide for Care and Use of Laboratory Animals” and the ethics and regulations of Shanghai University of Traditional Chinese Medicine (IACUC Issue No: SZY201711003).

The mice were randomly divided into six groups: normal group (N, distilled water 10 ml/kg), model group (M, distilled water 10 ml/kg), ASH high-dose group (HD, 400 mg/kg), middle-dose group (MD, 200 mg/kg), low-dose group (LD, 100 mg/kg) (Zhang et al., 2010), and positive control group (PD, fluoxetine, 10 mg/kg). According to the standard of Chinese Pharmacopoeia and the conversion standard of animal drugs, the dosages of different concentrations were prepared respectively and fluoxetine administration was performed according to the literature (Wang et al., 2015; Wan et al., 2017), and the solvent was distilled water. The mice were administered (*i.g.*) of ASH or fluoxetine from 9:30 to 10:30 (1 h before modeling) every day for 6 weeks. The administration started on the first day of modeling and continued throughout the modeling period.

#### 2.2.3 CUMS model

The specific operations of the CUMS method included food deprivation (24 h), water deprivation (24 h), overnight lighting (24 h), wet wood chips (24 h), tail pinching (1 min), tilting cage (24 h), and swimming in cold water at 4°C (5 min). All stresses did not threaten the lives of mice, and mice were randomly given different stresses every day to avoid adaptation. Animal welfare was strictly followed in accordance with the “Guidelines for the Care and Use of Laboratory Animals” and the regulation of Shanghai University of Traditional Chinese Medicine (IACUC Issue No: SZY201711003).

#### 2.2.4 Sample collection and processing

The liver tissues were collected and stored in the refrigerator at −80°C for further testing. A measure of 500°µL of the methanol–water–chloroform mixed solution was added to the centrifuge tube with a 50 mg liver sample. The samples were homogenized at 70 Hz for 80 s, vortexed for 1 min, sonicated for 5 min, and placed at −20°C for 20 min to precipitate the protein. Then the samples were centrifuged for 10 min (13,000 rpm, 4°C) and 300 µL supernatant was collected. A measure of 20 µL of methanolic heptadecanoic acid solution (1.0 mg/ml) was added to the supernatant. The supernatant was blown dry with nitrogen at 30°C and reconstituted with 50 µL of O-methoxyamine–HCl pyridine solution (15 mg/ml). The samples were transferred into a shaker for methoxylation reaction for 90 min (30°C). After the reaction, 50 µL of BSTFA was added and the silylation reaction was carried out for 1 h (70°C). After being placed at room temperature for 1 h, the samples were analyzed on the GC–MS.

#### 2.2.5 GC–MS analysis

The GC–MS column was Agilent J&W DB-5ms Ultra Inert (30 m × 0.25 mm × 0.25 μm). GC parameters: high-purity helium (purity: 99.9996%) was the carrier gas, the injection port temperature was 260°C with splitless injection and 1.0 µL injection volume, and the flow rate was 1.0 ml/min. The initial temperature was 80°C and lasted for 2 min. The temperature was raised to 180°C at a rate of 10°C/min, then to 240°C at 5°C/min, and finally to 290°C at 25°C/min and kept for 9 min. MS parameters: ion source temperature was 230°C, quadrupole temperature was 150°C, and mass spectrometer interface temperature was 280°C. Solvent delay was 5 min, ionization mode was EI, electron impact ionization voltage was 70 eV, and scan range (m/z) was 30–550. The parallel injection sequence method was used.

#### 2.2.6 Data processing

The raw data were imported into R software (v2.13.2) for data preprocessing. Subsequently, the processed data were imported into SIMCA software (v14.0, Umetrics AB, Umeå, Sweden) for multi-dimensional statistical analysis, including principal component analysis (PCA), partial least squares-discriminant analysis (PLS-DA), and orthogonal partial least squares-discriminant analysis (OPLS-DA). The S-plot was obtained on the basis of OPLS-DA, and variable importance in the projection (VIP) value >1.0 as the standard to find candidate difference variables. Then the value was imported into SPSS software (v21.0) for an independent sample t-test. *p* < 0.05 was defined as a significant difference, and fold change (FC) was calculated based on the average relative peak area of the difference variable between each group. Finally, values with VIP ≥1 and *p*-value ≤ 0.05 were determined as differential metabolites.

#### 2.2.7 Pathway analysis of metabolites and network construction

Combined with the Human Metabolome Database (HMDB, http://www.hmdb.ca/) to further confirm potential biomarkers, and MetaboAnalyst 4.0 (http://www.metaboanalyst.ca/) platform and Kyoto Encyclopedia of Genes and Genomes (KEGG, http://www.genome.jp/kegg/) database were used for enrichment analysis of metabolic pathways. Key pathways were determined with *p*-value < 0.05, and targets related to the pathways were identified based on the KEGG database.

### 2.3 Integrated analysis of network pharmacology and metabolomics

The overlapping targets were obtained from the targets of relevant pathways and the ASH related targets. These shared targets were potential targets for ASH treating MDD. Finally, the metabolite pathway target network was constructed by Cytoscape.

### 2.4 Molecule docking

The protein structure database Protein Data Bank (http://www.rcsb.org/) was used to obtain the structural information of the target. The docking software LigPlot (https://www.ebi.ac.uk/thornton-srv/software/LigPlus/) was used to make images of the interaction between protein molecules and ligand molecules.

## 3 Results

### 3.1 Screening the compounds and targets of ASH

Through the literature search, a total of 20 main compounds in ASH were retrieved (Yu et al., 2003; Du and Zhao, 2008; Gong and Wang, 2012; Lu et al., 2012; Yang et al., 2015). The OB and DL of these compounds were obtained by the TCMSP database, and the results are shown in Table 1. From the UniProt database, the gene names were identified, the invalid and duplicate targets were removed, and finally, 281 targets were obtained.

**TABLE 1:** Information of active compounds in ASH.

Molecule	OB (%)	DL	GI absorption	BBB	Reference
Protocatechuic acid	25.37	0.04	High	No	Shang et al. (2018)
Chlorogenic acid	11.93	0.33	Low	No	Yu et al. (2003)
Rutin	3.2	0.68	Low	No	Gong and Wang (2012)
Hyperoside	6.94	0.77	Low	No	Gong and Wang (2012)
Quercetin	46.43	0.28	High	No	Gong and Wang (2012)
Quercitrin	4.04	0.74	Low	No	Gong and Wang (2012)
Daucosterol (eleutheroside A)	20.63	0.63	Low	No	Du and Zhao (2008)
Syringin (eleutheroside B)	14.64	0.32	Low	No	Lu et al. (2012)
Eleutheroside B1	-	-	Low	No	Du and Zhao (2008)
Eleutheroside C	-	-	High	No	Du and Zhao (2008)
Eleutheroside D	-	-	Low	No	Du and Zhao (2008)
Eleutheroside E	16.85	0.29	Low	No	Du and Zhao (2008)
Sesamin	56.55	0.83	High	Yes	Zhang and Li (2016)
Isofraxidin	52.32	0.1	High	Yes	Gong and Wang (2012)
Kaempferol	41.88	0.24	High	No	Gong and Wang (2012)
Acacetin	34.97	0.24	High	No	Gong and Wang (2012)
Daidzin	14.32	0.73	Low	No	Gong and Wang (2012)
Syringaldehyde	67.06	0.05	High	Yes	Gong and Wang (2012)
Syringic acid	47.78	0.06	High	No	Gong and Wang (2012)
Glucosyringic acid	24.29	0.3	Low	No	Gong and Wang (2012)

Note: - means no information about this compound. OB, oral bioavailability; DL, drug-likeness; GI, gastrointestinal; BBB, blood–brain barrier.

### 3.2 MDD target and network identification

**FIGURE 2:**
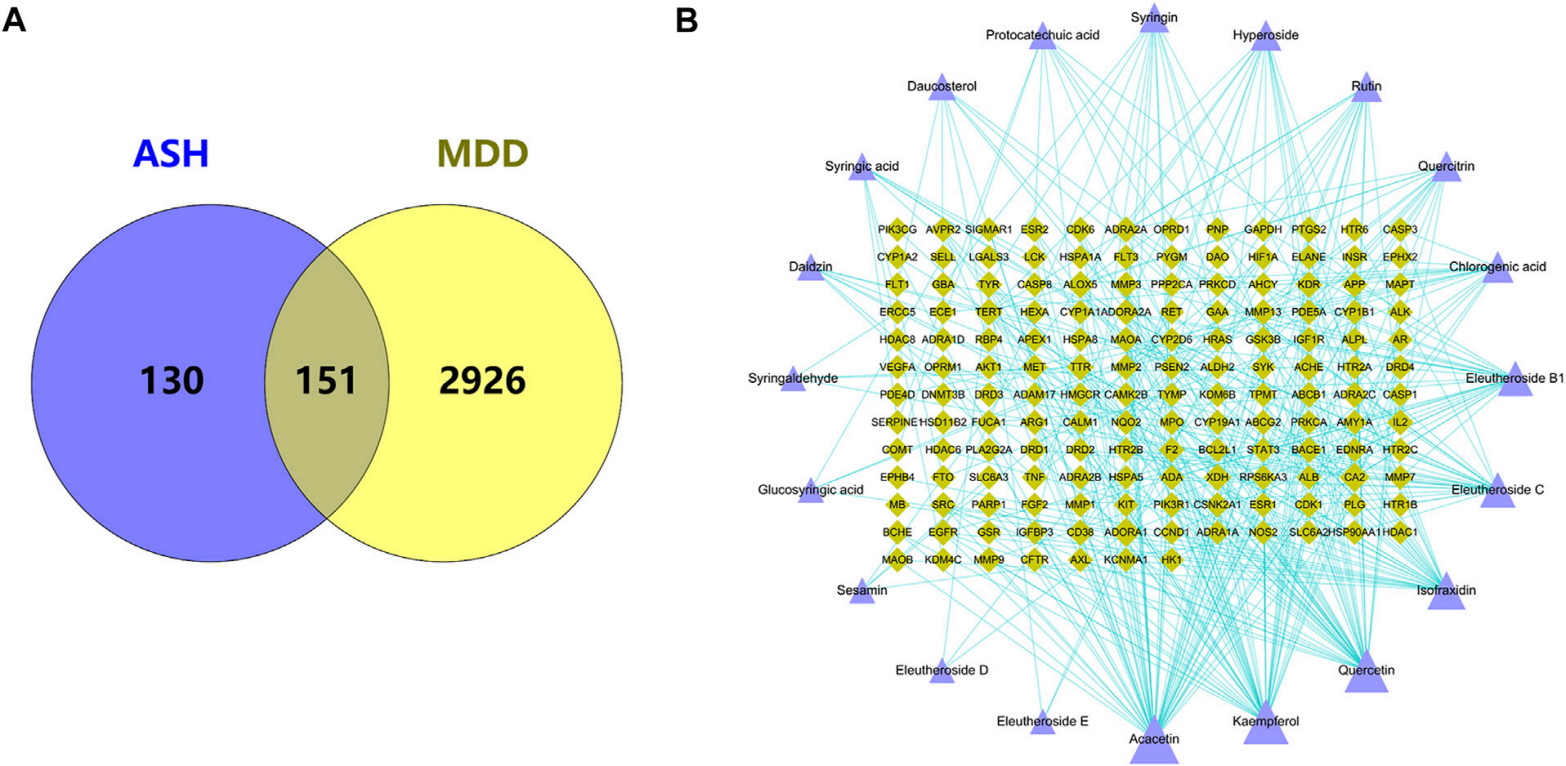
**(A)** Overlapping target genes between MDD and ASH. **(B)** Drug-active ingredients and disease target network. Purple triangle: active compounds; green diamond: targets. The bigger nodes represent greater degrees.

Through the integration of the results of each database, 3077 MDD-related gene targets were obtained. Using the Venn diagram, 151 shared targets of ASH and MDD were obtained (Figure 2A). Then we used the targets related to ASH and corresponding compounds to build a network and visualized it using Cytoscape (Figure 2B). The network has 171 nodes (20 compounds of ASH and 151 shared targets) and 361 edges.

### 3.3 Analysis of potential biomarkers

First of all, as shown by the behavioral test results in Supplementary Figure S2, the immobility time of the mice in the CUMS model group was significantly increased in the FST and the TST, and the total behavioral score in the OFT was significantly decreased. After administration of different doses of ASH and the positive control fluoxetine, all behavioral scores were significantly improved, and the effects of middle-dose ASH were relatively most significant. These confirmed a successful depression model and antidepressant effect of ASH.

As shown in Supplementary Figures S3A,B, the overall metabolic profiles of the liver of mice in the N and M groups only showed a tendency to separate in the PCA score. The PLS-DA score was further performed and the results showed that the separation trend of the metabolic profiles of the N group and the M group was more obvious.

The metabolic patterns of the ASH high, medium, and low-dose groups and fluoxetine group are shown in Supplementary Figures S3C–F. In the PCA analysis, the N group and the M group showed a trend of separation, and the HD group, MD group, and LD group were all close to the N group, of which the most obvious one was the MD group. The PD group was also significantly separated from the M group, closer to the N group. The results indicated that each dose of ASH and fluoxetine could restore the hepatic metabolism in CUMS mice to some extent.

**FIGURE 3:**
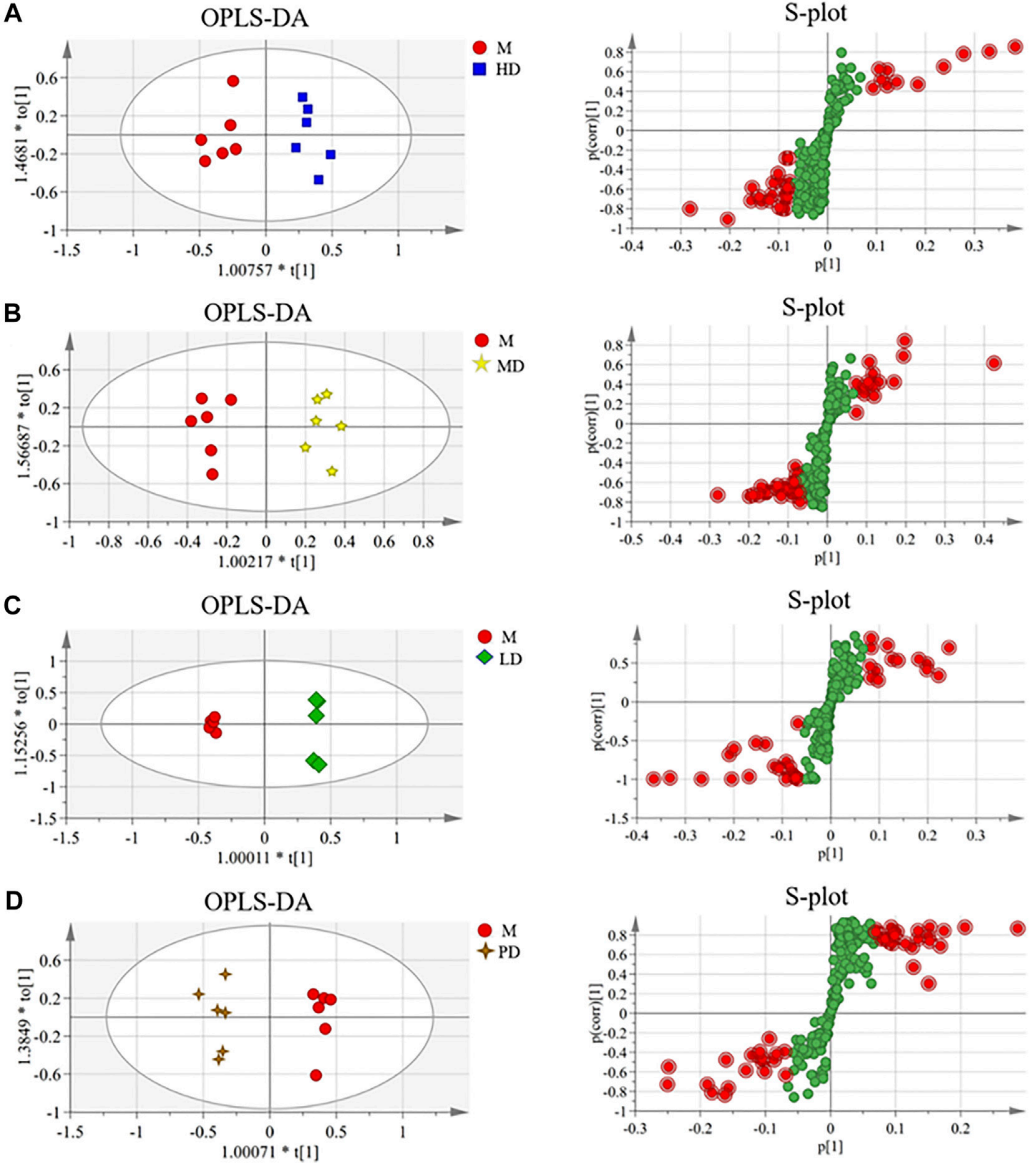
OPLS-DA scores and S-plot of the liver metabolite group of CUMS mice in each administration group (*n* = 6). **(A)** HD group vs. M group (R^2^X = 0.655, R^2^Y = 0.927, and Q^2^ = 0.568); **(B)** MD group vs. M group (R^2^X = 0.759, R^2^Y = 0.959, and Q^2^ = 0.422); **(C)** LD group vs. M group (R^2^X = 0.762, R^2^Y = 0.999, and Q^2^ = 0.968); **(D)** PD group vs. M group (R^2^X = 0.777, R^2^Y = 0.978, and Q^2^ = 0.664).

In order to find the different metabolites between each dose group of ASH and the M group, the OPLS-DA and S-plot score under the supervision mode were established (Figure 3). In the OPLS-DA analysis, the HD group, the MD group, LD group, and PD group were all separated from the M group. The results of the permutation test (Supplementary Figure S4) showed that the established model was not overfitting. The variables with VIP value > 1.0 were selected for statistical analysis, which was combined with the NIST 05 database and HMDB database to get the final differential metabolites.

**FIGURE 4:**
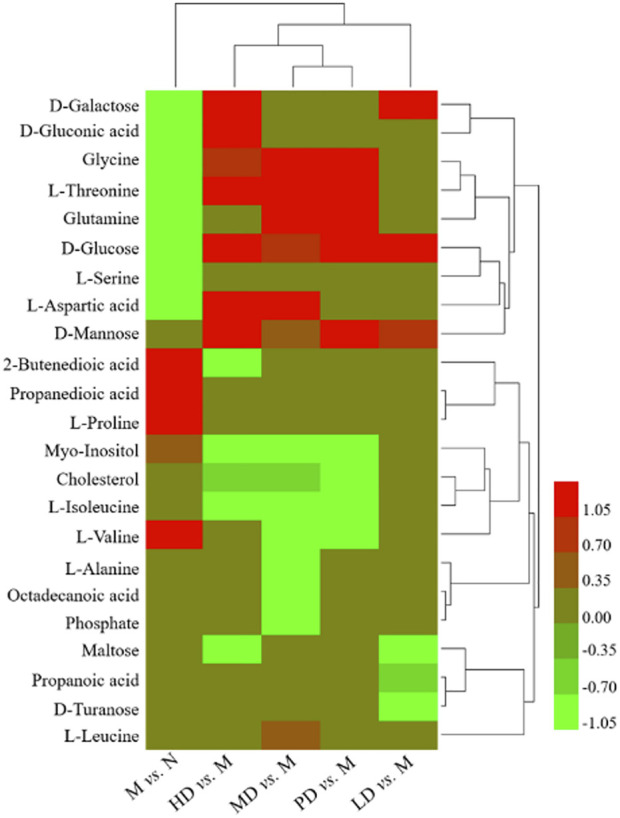
Heatmap of different metabolites in each group.

As shown in Table 2, the levels of eight metabolites improved in the HD group. Compared with the M group, the levels of D-galactose, D-gluconic acid, glycine, D-glucose, L-aspartic acid, and L-threonine were significantly increased, while 2-butenedioic acid and myo-inositol levels were significantly reduced. The levels of five metabolites were increased in the MD group, glycine, D-glucose, L-aspartic acid, L-threonine, and glutamine, while the levels of myo-inositol and L-valine were significantly decreased. D-galactose and D-glucose were improved in the LD group. In the PD group, the levels of glycine, D-glucose, L-threonine, and glutamine were significantly increased, and the levels of myo-inositol and L-valine were significantly decreased. The heatmap of differential metabolites is shown in Figure 4, which indicated the differential metabolites among each group.

**TABLE 2:** Intervention of ASH and fluoxetine on different metabolites in the liver of CUMS mice.

Metabolite	M vs. N		HD vs. M		MD vs. M		LD vs. M		PD vs. M	
	*P*	FC^a^	*P*	FC^b^	*P*	FC^b^	*P*	FC^b^	*P*	FC^b^
d-Galactose	0.007	0.185	0.002	6.143	-	-	<0.001	2.299	-	-
d-Glucose	0.016	0.132	0.007	7.547	0.02	1.951	0.001	2.314	0.010	2.152
Maltose	-	-	0.015	2.291	-	-	<0.001	0.006	-	-
d-Mannose	-	-	0.034	2.331	0.038	1.276	0.001	1.641	0.002	2.294
d-Turanose	-	-	-	-	-	-	<0.001	0.002	-	-
d-Gluconic acid	0.012	0.145	0.011	8.799	-	-	-	-	-	-
2-Butenedioic acid	<0.001	3.590	0.005	0.340	-	-	-	-	-	-
Propanedioic acid	0.006	3.402	-	-	-	-	-	-	-	-
Octadecanoic acid	-	-	-	-	0.003	0.325	-	-	-	-
Phosphate	-	-	-	-	0.010	0.367	-	-	-	-
Propanoic acid	-	-	-	-	-	-	0.014	0.681	-	-
Glycine	0.015	0.602	0.009	1.906	0.009	2.242	-	-	<0.001	3.306
l-Serine	0.002	0.244	-	-	-	-	-	-	-	-
l-Aspartic acid	0.015	0.157	0.024	4.585	0.028	6.463	-	-	-	-
l-proline	0.033	4.040	-	-	-	-	-	-	-	-
l-Leucine	-	-	-	-	0.011	1.342	-	-	-	-
l-Alanine	-	-	-	-	0.032	0.307	-	-	-	-
l-Threonine	0.003	0.256	0.016	2.787	0.009	3.691	-	-	<0.001	13.143
l-Valine	0.013	6.254	-	-	0.019	0.303	-	-	0.011	0.064
Glutamine	0.009	0.270	-	-	0.029	5.315	-	-	0.011	10.233
l-Isoleucine	-	-	0.025	0.364	0.028	0.394	-	-	0.002	0.146
Cholesterol	-	-	0.001	0.632	0.013	0.694	-	-	<0.001	0.473
Myo-inositol	0.038	1.382	0.049	0.591	0.008	0.528	-	-	0.008	0.490

Note: Fold change (FC)^a^ represents the change multiple of the relative content of the different metabolites in the M group compared to the N group (FC^a^, value = M/N). FC^b^ represents the change multiple of the relative content of the different metabolites in the administration group compared to the M group (FC^b^ value = the administration group/M). - means that the compound is not detected. FC, fold change.

### 3.4 Metabolic pathways and relevant target verification

**FIGURE 5:**
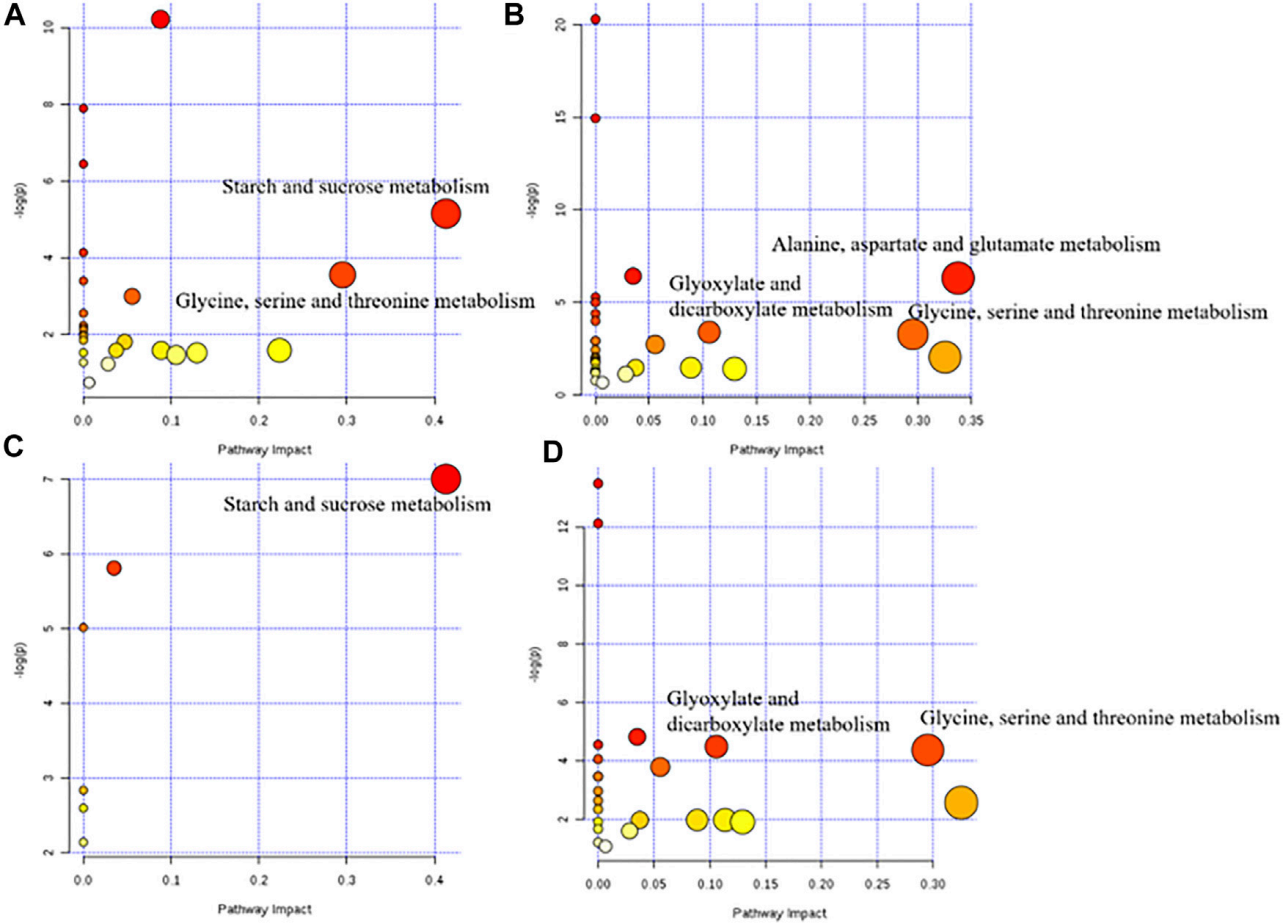
Metabolic pathway diagram of each administration group. **(A)** HD group vs. M group; **(B)** MD group vs M group; **(C)** LD group vs. M group; **(D)** PD group vs. M group.

The metabolic pathways between the model group and each dose of the ASH group are shown in Figure 5. According to statistical analysis, the metabolic pathways with *p* < 0.05 and pathway impact >0.10 were selected.

**FIGURE 6:**
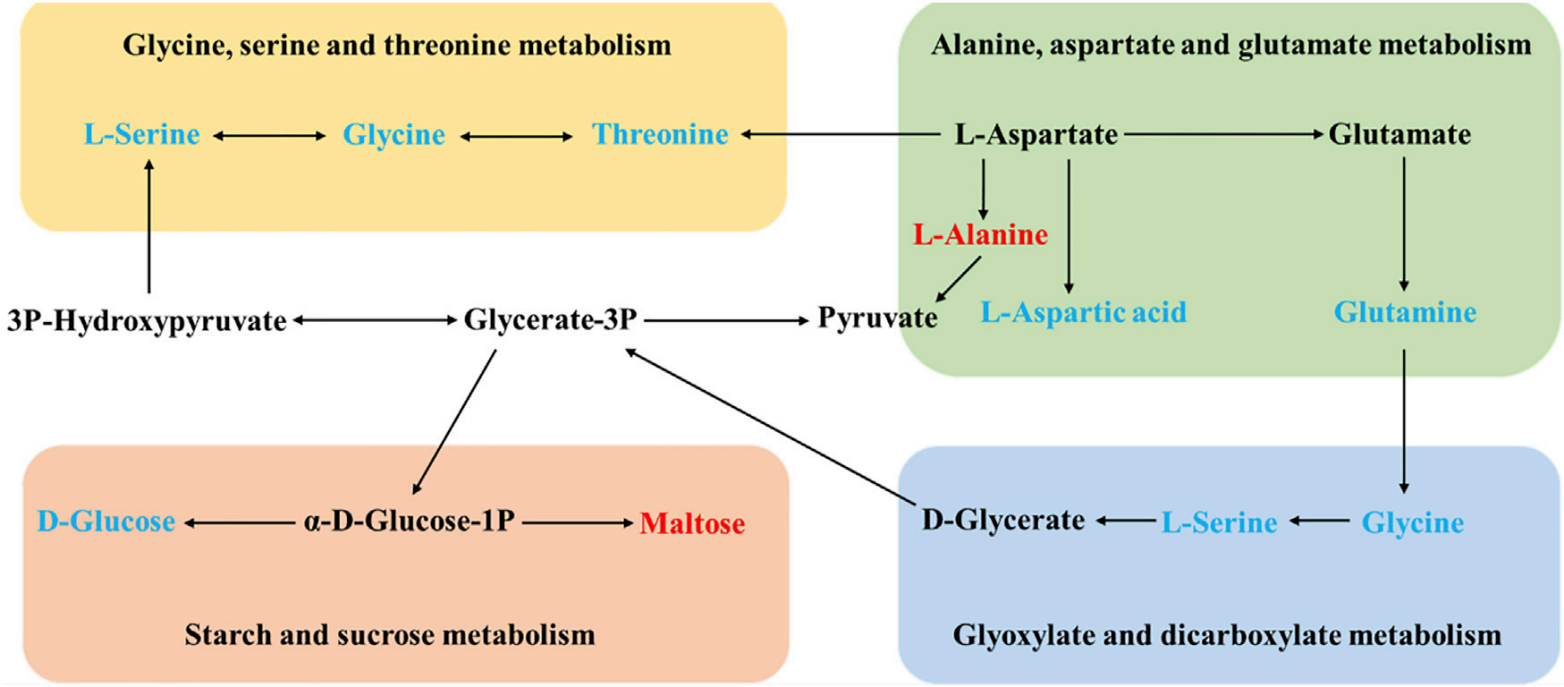
Schematic diagram of the metabolic pathways. In the model group, red and blue metabolites represent increased and decreased levels, respectively.

The differential metabolite between the model group and the HD group were involved in two pathways, glycine, serine, and threonine metabolism and starch and sucrose metabolism. There were three metabolic pathways between the model group and the MD group, glyoxylate and dicarboxylate metabolism; glycine, serine, and threonine metabolism; and alanine, aspartate, and glutamate metabolism. Differential metabolites between the model group and the LD group only constitute one metabolic pathway, which is starch and sucrose metabolism. Also, two pathways of glyoxylate and dicarboxylate metabolism and glycine, serine, and threonine metabolism were corresponding for differential metabolites between the model group and the PD group. The differential metabolites involved in these metabolic pathways are shown in Table 3. Based on these results, and combined with the KEGG database, the key metabolic pathways of MDD are illustrated in Figure 6.

**TABLE 3:** Intervention of ASH and fluoxetine on liver metabolic pathways in CUMS mice.

Group	Metabolite pathway	*p-*value	Pathway impact	Related metabolite
M vs. N	Aminoacyl-tRNA biosynthesis	3.18E-08	0.167	l-Serine, glycine, glutamine, l-valine; l-aspartic acid, l-threonine, l-proline
	Glycine, serine, and threonine metabolism	0.003	0.501	l-Serine, glycine, l-threonine
	Alanine, aspartate, and glutamate metabolism	0.023	0.337	Glutamine, l-aspartic acid
	Glyoxylate and dicarboxylate metabolism	0.002	0.148	l-Serine, glycine, glutamine
HD vs. M	Glycine, serine, and threonine metabolism	0.028	0.295	Glycine, l-threonine
	Starch and sucrose metabolism	0.006	0.413	d-Glucose, maltose
MD vs. M	Glyoxylate and dicarboxylate metabolism	0.034	0.106	Glycine, glutamine
	Glycine, serine, and threonine metabolism	0.034	0.295	Glycine, l-threonine
	Alanine, aspartate, and glutamate metabolism	0.002	0.337	Glutamine, l-alanine, l-aspartic acid
LD vs. M	Starch and sucrose metabolism	0.001	0.413	d-Glucose, maltose
PD vs. M	Glyoxylate and dicarboxylate metabolism	0.011	0.106	Glycine, glutamine
	Glycine, serine, and threonine metabolism	0.013	0.295	Glycine, l-threonine

Based on the aforementioned results, the following four pathways, glycine, serine, and threonine metabolism; alanine, aspartate, and glutamate metabolism; glyoxylate and dicarboxylate metabolism; and starch and sucrose metabolism, were selected to obtain targets with the KEGG database. There were 130 targets in total.

### 3.5 Integrating analysis

**FIGURE 7:**
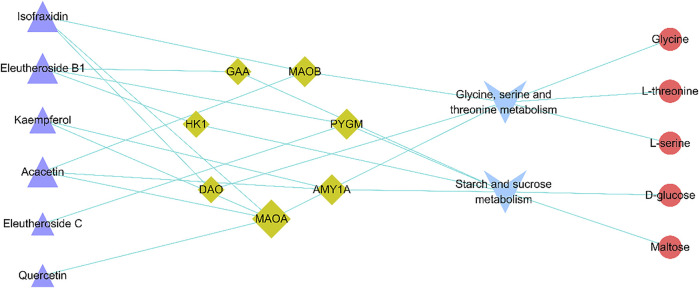
Active compound–overlapping target-metabolic pathway potential biomarker interaction network. Purple triangle: active compounds; green diamond: targets; blue V: metabolic pathways; red circle: potential biomarkers. The bigger nodes represent greater degrees.

**FIGURE 8:**
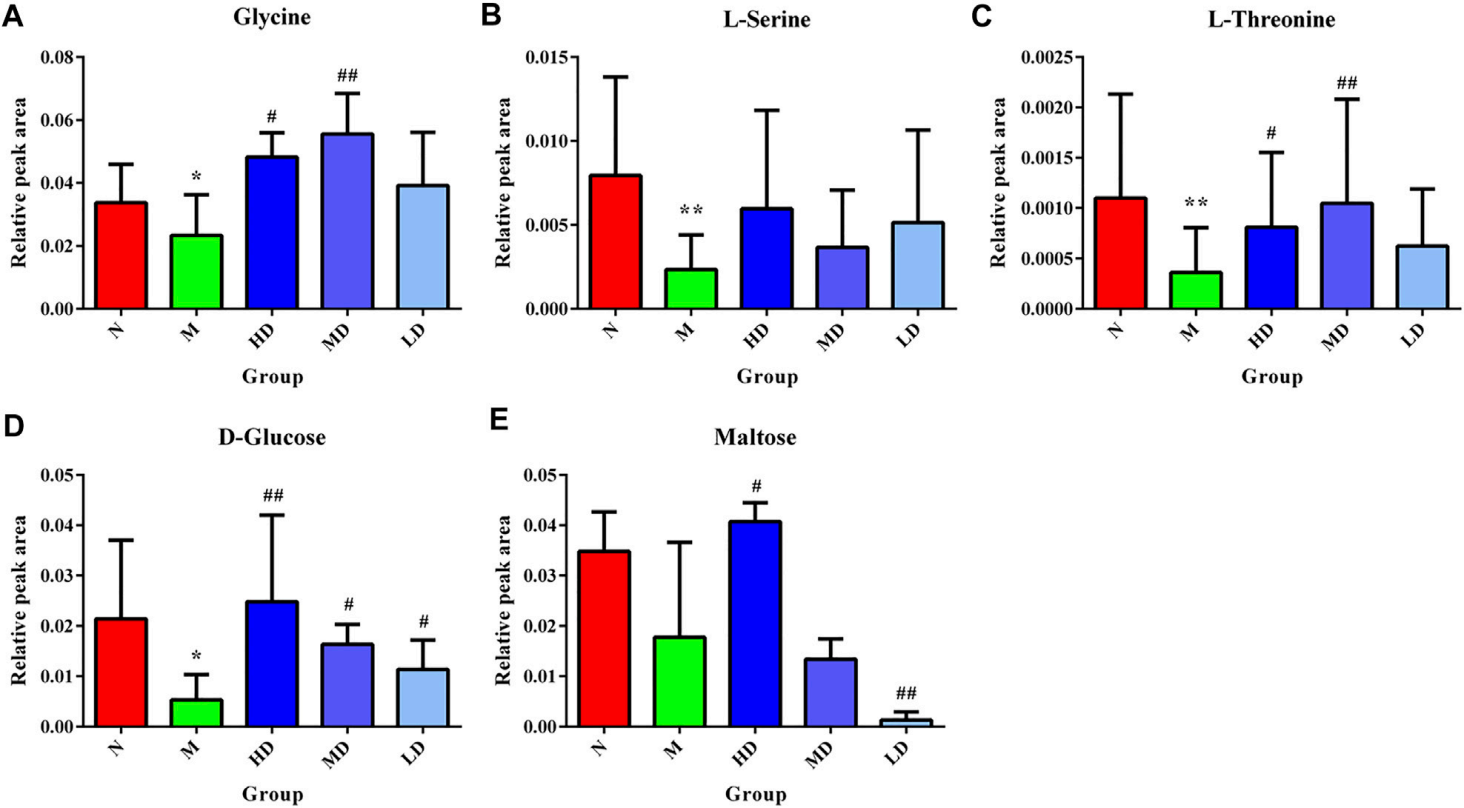
Relative content of the hepatic biomarkers in each group. **(A)** Glycine; **(B)** l-serine; **(C)** l-threonine; **(D)** d-glucose; **(E)** maltose. *p < 0.05, compared with the N group; **p < 0.01, compared with the N group; ^#^ *p* < 0.05, compared with the M group; ^##^ *p* < 0.01, compared with the M group.

Integrating 130 targets involved in four pathways and the targets related to compounds in ASH, seven shared targets related to two pathways that were glycine, serine, and threonine metabolism and starch and sucrose metabolism were identified, which were d-amino acid oxidase (DAO), monoamine oxidase A (MAOA), monoamine oxidase B (MAOB), alpha glucosidase (GAA), amylase alpha 1A (AMY1A), hexokinase 1 (HK1), and glycogen phosphorylase muscle associated (PYGM). The Cytoscape was applied to visualize the network of compounds in ASH, shared targets, pathways, and relevant metabolites (Figure 7). In addition, from the network, glycine, serine, and threonine metabolism was associated with three metabolites, glycine, L-serine, and L-threonine, while D-glucose and maltose were related to starch and sucrose metabolism. The changes in relative peak areas of five metabolites in each group are shown in Figure 8. In untargeted metabolomics studies, the relative peak area is often used to represent the relative content of the metabolite to which the peak belongs. It is the ratio of the peak area of each metabolite to the peak area of the internal standard in the same sample. Each sample was added the same known amount of the internal standard compound in the procedure of sample pretreatment. Through the ratio to the peak of the internal standard compound, the systematic errors caused by pretreatment or analytical instrument can be reduced.

### 3.6 Molecule docking

To further verify the affinity of the overlapped targets and the relevant compounds, molecular docking was carried out for these compounds with the related targets of each active ingredient, including isofraxidin docking with DAO, MAOA, and MAOB; quercetin docking with MAOA; kaempferol docking with MAOA; acacetin docking with MAOA and MAOB; eleutheroside B1 docking with GAA, HK1, and PYGM; eleutheroside C docking with PYGM.

The binding energies of the six compounds to the relevant targets were calculated. The results are listed in Supplementary Table S1. All the studied compounds had good binding energy (<0 kcal/mol) to their relevant target proteins. Binding energy <0 indicated that the ligand molecular compounds could bind autonomously to the receptor target protein. The molecule conformation with a lower binding energy was more stable.

**FIGURE 9:**
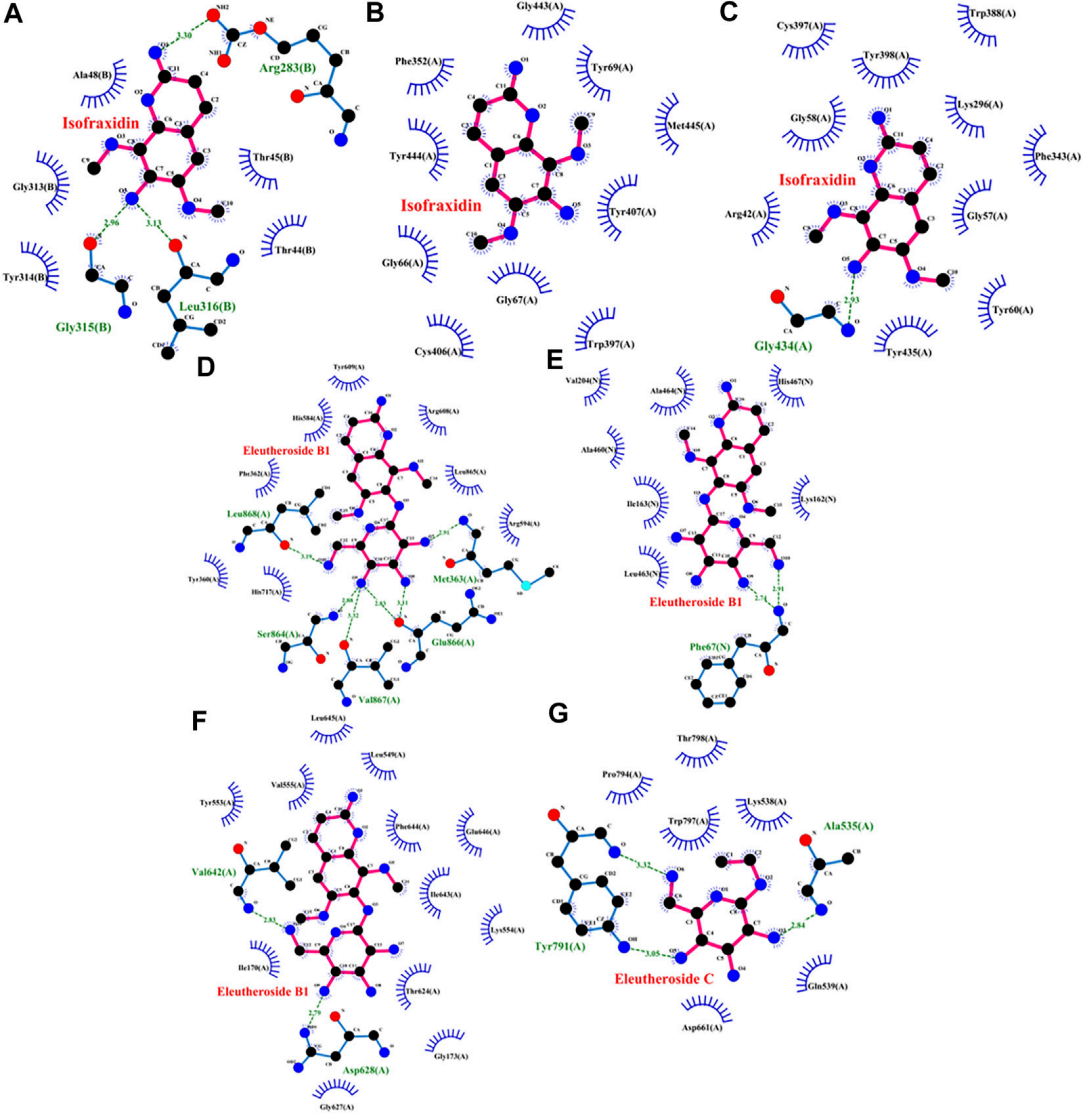
Molecular docking charts of isofraxidin [DAO **(A)**, MAOA **(B),** and MAOB **(C)**], eleutheroside B1 [GAA **(D)**, HK1 **(E)**, and PYGM **(F)**], and eleutheroside C [PYGM **(G)**].

As shown in the Figures 9A–C, isofraxidin had three hydrogen bonds and five hydrophobic interactions with DAO, 10 hydrophobic interactions with MAOA and 1 hydrogen bond and 10 hydrophobic interactions with MAOB. Eleutheroside B1, as shown in Figures 9D,E, had six hydrogen bonds and eight hydrophobic interactions with GAA as well as two hydrogen bonds and seven hydrophobic interactions with HK1. The protein PYGM, as shown in Figures 9F,G, interacted with eleutheroside B1 creating two hydrogen bonds and 12 hydrophobic interactions, while interacted with eleutheroside C creating three hydrogen bonds and six hydrophobic interactions. In addition, quercetin, kaempferol, and acacetin also had a good combination with their corresponding targets (Supplementary Figure S5).

## 4 Discussion

In the metabolomics analysis, glycine, serine, and threonine metabolism; alanine, aspartate, and glutamate metabolism; glyoxylate and dicarboxylate metabolism; and starch and sucrose metabolism were the most significant pathways for ASH treating MDD, and a total of 130 targets were involved with the four metabolic pathways, including DAO, MAOA, MAOB, GAA, HK1, and PYGM. Network pharmacology analysis has shown that the intersection of ASH targets and MDD targets was 151, which also includes DAO, MAOA, MAOB, GAA, HK1, and PYGM. Therefore, integrating metabolomics and network pharmacology together, DAO, MAOA, MAOB, GAA, HK1, and PYGM were shared targets in both results. Finally, molecule docking showed that all these targets could dock stably with their corresponding compounds, including isofraxidin, eleutheroside B1, eleutheroside C, quercetin, kaempferol, and acacetin. In summary, ASH influenced the pathological performance of MDD through the two pathways, glycine, serine, and threonine metabolism and starch and sucrose metabolism, and targets DAO, MAOA, MAOB, GAA, HK1, and PYGM.

The remarkable pathway, glycine, serine, and threonine metabolism obtained from the results of metabolomics, is commonly seen in the mental disorders (Yang et al., 2020). Similar to our results, glycine levels in patients with depression were decreased (Zhao et al., 2015; Li et al., 2019). Glycine and serine are neurotransmitters (Opladen et al., 2016). Glycine is proven to have an anti-atherosclerotic effect (Grajeda-Iglesias and Aviram, 2018), and low glycine levels in patients with MDD may mean that MDD patients have a risk of atherosclerosis (Hung et al., 2021). Threonine is an indispensable amino acid to the nervous system. It can be converted to glycine and transferred through the blood–brain barrier (Bränn et al., 2021). Excessive threonine in the nervous system would affect the balance of neurotransmitters (Boehm et al., 1998). Serine, a non-essential amino acid, is actively transported to the brain and then converted into glycine and phosphatidylcholine, both of which are related to memory function (Woronczak et al., 1995; Locasale, 2013). Both serine and glycine regulate N-methyl-D-aspartate (NMDA) receptors, which play a major role in the glutamate pathway in the brain (van den Brink et al., 2017). The DA and glutamate systems in the brain are highly interrelated (Javitt, 2007).

From the results of network pharmacology, targets DAO, MAOA, and MAOB were involved in the glycine, serine, and threonine metabolism. DAO, MAOA, and MAOB are widely distributed in the liver and kidney, and DAO is mainly in the intestine. DAO is a peroxisomal flavoprotein, which can directly affect the level of serine. The research found that excessive DAO expression would exacerbate schizophrenia (Labrie et al., 2010). Serine is abundant in the forebrain and acts as a co-agonist of NMDA receptors to enhance neurotransmission. At the same time, DAO can catabolize serine and, therefore, modulate neurotransmission (Yamanaka et al., 2012). Isofraxidin has a good docking with DAO, suggesting that isofraxidin may alleviate depression symptoms by regulating DAO.

Monoamine oxidases (MAOs) have two subtypes: MAOA and MAOB, which are thought to mediate the degradation of monoamine neurotransmitters (including DA) in the brain. Cho et al. (2021) found that MAOA could regulate DA levels, whereas MAOB could control tonic GABA levels. Also, elevated MAO activity is responsible for inactivation of monoamine neurotransmitters in neurological diseases, such as depression (Singh et al., 2020). MAO inhibitor, one of the first discovered antidepressants, reduces the degradation of central monoamine neurotransmitters (such as 5-HT and DA) by inhibiting MAO, and improves patient mood. In our results of molecular docking, isofraxidin, quercetin, acacetin, and kaempferol were all bound well to MAOA and MAOB, suggesting that the mechanism of antidepressants of these compounds is similar to MAO inhibitors. Also, similar to our results, He et al. (2020) found from the root extract of ASH that isofraxidin had potential inhibitory activity of MAOB ligand. At the same time, studies have shown that some flavonoids (including quercetin, acacetin, and kaempferol) had inhibitory activity of MAOA or MAOB (Dhiman et al., 2019; Xiao et al., 2019; Lin et al., 2020).

Starch and sucrose metabolism is another major pathway in the results of our study, and it is a prominent pathway in patients with MDD (Chung et al., 2019). It belongs to carbohydrate metabolism, and the main different metabolites involved in our study are D-glucose and maltose. Maltose is a disaccharide composed of two glucose units connected by glycosidic bonds. Glucose is the main source of energy, and in our results, D-glucose was decreased in the model group, implying disturbed energy metabolism. Also, energy deficiency leads to the most common depressive symptoms, including decreased activity, physical fatigue, and slowed cognitive function (Ren et al., 2020). Abnormal glucose metabolism in depression is proven to be associated with suicide risk, which may be related to the cytokine-mediated inflammatory process (Koponen et al., 2015). Glucose metabolism homeostasis is the basis for maintaining normal brain function, and in MDD patients, the glucose metabolism in many areas of the brain is reduced (Hundal, 2007; Fu et al., 2017). In depression or obesity models of depression, the uptake of glucose by brain cells is enhanced, or in other words, its metabolism is slower (Detka et al., 2014). At the same time, glucose is the main energy substrate of neurons and glial, which is very important to the neuron microenvironment (Magistretti and Pellerin, 1999).

GAA, acid *α*-glucosidase, is widely distributed in the systemic circulation and can cause glycogen accumulation in many tissues and the entire central nervous system and severe neuromuscular damage when deficient, which eventually leads to Pompe disease, a metabolic and neuromuscular disorder (In't Groen Stijn et al., 2020). Increased GAA expression in the liver can improve glycogen accumulation in the muscles and central nervous system (Puzzo et al., 2017). In the brain, glycogen is an important energy reserve, and the reduction of the glycogen level is directly related to the metabolism and function of astrocytes (Zhang et al., 2015). In particular, the main energy substrate source of neurons is glucose, which enters astrocytes and produces stored glycogen. When astrocytes cannot provide glycogen to the brain in time, neurons will shrink and die. If the glycogen content in the central nervous system is reduced, neurotransmitters and action potentials will be severely affected immediately (Ibrahim et al., 2011). The study has shown that a low level of hippocampal glycogen may be one of the mechanisms that induce depression-like behavior in mice (Zhang et al., 2015).

HK1, mainly distributed in the brain, is a kind of hexokinase that initiates the first step of glycolysis by the phosphorylation of glucose (JE, 2003). HK1 attached to the outer mitochondrial membrane (OMM) is one of the crucial features of brain energy metabolism, and also prevents apoptosis and oxidative damage, which ensures the survival of neurons and other cells (Regenold et al., 2012). A study has found in postmortem parietal cortex brain tissue with depression, a decrease in HK1 attachment to the OMM and schizophrenia compared to the health controls (Regenold et al., 2012). In addition, HK1 mitochondrial attachment has also been linked to neural growth (Wang et al., 2008) and brain development (Land et al., 1977). The survival and growth of neurons involved in mood and cognitive functions are critical to the treatment of depression (Cristy, 2017). Also, a series of infections or just simply inflammation during pregnancy may increase the risk of autism and depression in the child (Al-Haddad et al., 2019).

PYGM, a muscle glycogen phosphorylase or myophosphorylase, is mainly involved in glycogenolysis and provides sufficient energy for cell biological processes (Migocka-Patrzałek and Magdalena, 2021). PYGM is highly expressed in human skeletal muscle, but it is also present in other tissues and organs, such as different parts of the brain, liver, lymphatic tissue (tonsils), blood (granulocytes), salivary glands, and adipose tissue (Uhlén et al., 2015). Lack of PYGM in the liver can cause Hers disease, a glycogen-storage disease (Burwinkel et al., 1998). The research has found that in the astrocytes of schizophrenia, the levels of PYGM and RAC1 (a kinase that regulates the activity of PYGM) involved in astrocytes metabolism are reduced, leading to a transient partial energy deficiency in the dorsolateral prefrontal cortex (Pinacho et al., 2016). In the dorsolateral prefrontal cortex, glutamate-mediated neurotransmission disorders and changes in energy metabolism are commonly observed in schizophrenia (Uno and Coyle, 2019; Sears and Auid-Orcid, 2021). RAC1 promotes glycogenolysis by activating PYGM and provides instant energy for neurons. Also, this source of energy is essential for the processes of glutamatergic neurotransmission and glucose utilization (Migocka-Patrzałek and Magdalena, 2021). The hypothesis of glutamate-mediated neurotransmission disorder has gradually become popular in depression research in recent years. A vast majority of brain neurons and synapses are glutamatergic, and glutamate synaptic transmission mainly mediates cognition and emotion (Pessoa, 2008). Decreased levels of glutamatergic metabolites have been observed in the medial frontal cortex of MDD patients (Moriguchi et al., 2019). Therefore, for the neuropsychiatric disease treatment, the regulation of glycogenolysis may be crucial.

In the results of molecular docking, eleutheroside B1 was bound well to GAA, HK1, and PYGM, while eleutheroside C had a good docking with PYGM. Therefore, we proposed that GAA and PYGM might affect the occurrence and development of depression by regulating glycogen metabolism in the brain, while HK1 affects the survival and growth of neurons by regulating glycolysis, and eventually affects the mood of depressed patients. Also, it was implied that eleutheroside B1 and eleutheroside C play an antidepressant effect by regulating energy metabolism.

Our study provides clues for the mechanism and material basis of ASH treatment of depression, but the current research still has some limitations. First of all, this study is based on the hepatic metabolomics study. Although hepatic metabolites may also enter the blood circulation and interact with the target or drug-active components, they still need to be compared with components in blood circulation and brain. Second, only part of the active components of ASH can be directly absorbed and pass through the blood–brain barrier. Although they are closely related to many depression targets in the prediction of network pharmacological targets, their actual binding sites in the body are still unclear. Also, based on the current results, it is speculated that the liver may play an important role in the antidepressant effect of ASH, and the targets may not be limited to the central nervous system. Finally, the current findings are still preliminary, and further validation is under design, such as the effect of administration of a single ingredient of ASH on depression and changes in distribution and expression of the predicted targets, as well as targeted, quantitative experiments for validating the role of those targets in the regulation of MDD by ASH.

## 5 Conclusion

In our study, first, ASH administration improved depression-like behaviors and simultaneously ameliorated hepatic metabolomic alterations in CUMS mice. Second, combined with network pharmacology and molecular docking techniques, the potential active components, targets, and related metabolic pathways of ASH in the treatment of depression were predicted, that is, isofraxidin, quercetin, kaempferol, and acacetin might target DAO, MAOA, and MAOB to regulate glycine, serine, and threonine metabolism, while eleutheroside B1 and eleutheroside C seemed to regulate starch and sucrose metabolism by targeting GAA, HK1, and PYGM. As the components in ASH and their targets are primarily derived from the literature and network pharmacology rather than direct experiments, these conclusions are predictive and require validation by both qualitative and quantitative experiments on compounds to single targets. In addition, there is a certain gap between the metabolic pathway results derived from the depression model mice and the clinical patients, so validation studies based on the quantitative analysis of metabolites in clinical samples are needed.

## Data Availability

The original contributions presented in the study are included in the article/Supplementary Material; further inquiries can be directed to the corresponding authors.

## Glossary

5-HT: 5-hydroxytryptamine
AMY1A: amylase alpha 1A
ASH: *Acanthopanax senticosus* (Rupr. et Maxim.) Harms
BBB: blood–brain barrier
CUMS: chronic unpredictable mild stress
DA: dopamine
DAO: D-amino acid oxidase
DL: drug-likeness
FC: fold change
FST: forced swimming test
GC–MS: gas chromatography–mass spectrometry
GAA: alpha glucosidase
GI: gastrointestinal
HK1: hexokinase 1
HPA: hypothalamus–pituitary–adrenal
HPLC: high-performance liquid chromatography
MAOA: monoamine oxidase A
MAOB: monoamine oxidase B
MDD: major depressive disorder
NE: norepinephrine
NMDA: N-methyl-D-aspartate
OB: oral bioavailability
OMM: outer mitochondrial membrane
OPLS-DA: orthogonal partial least squares-discriminant analysis
PCA: principal component analysis
PLS-DA: partial least squares-discriminant analysis
PYGM: glycogen phosphorylase muscle associated
SPT: sucrose preference test
TST: tail suspension test
VIP: variable importance in projection
