# Supplementary material for: Integrated network pharmacology and hepatic metabolomics to reveal the mechanism of Acanthopanax senticosus against major depressive disorder
Source: Front Cell Dev Biol. 2022 Aug 5;10:900637. doi: 10.3389/fcell.2022.900637 (PMC9389016; doi:10.3389/fcell.2022.900637)
Supplement: Supplementary file 1 [file Table1.DOCX]

**Table S1** Information of the binding energy of the compound and the corresponding target

| ID | MOL | Targets | Affinity (kcal/mol) |
| --- | --- | --- | --- |
| docking01 | Isofraxidin | DAO | -7.2 |
| docking02 | Isofraxidin | MAOA | -7.0 |
| docking03 | Quercetin | MAOA | -9.0 |
| docking04 | Kaempferol | MAOA | -8.8 |
| docking05 | Acacetin | MAOA | -9.3 |
| docking06 | Isofraxidin | MAOB | -7.3 |
| docking07 | Acacetin | MAOB | -9.0 |
| docking08 | Eleutheroside B1 | GAA | -7.2 |
| docking09 | Eleutheroside B1 | HK1 | -6.3 |
| docking10 | Eleutheroside B1 | PYGM | -6.9 |
| docking11 | Eleutheroside C | PYGM | -5.5 |


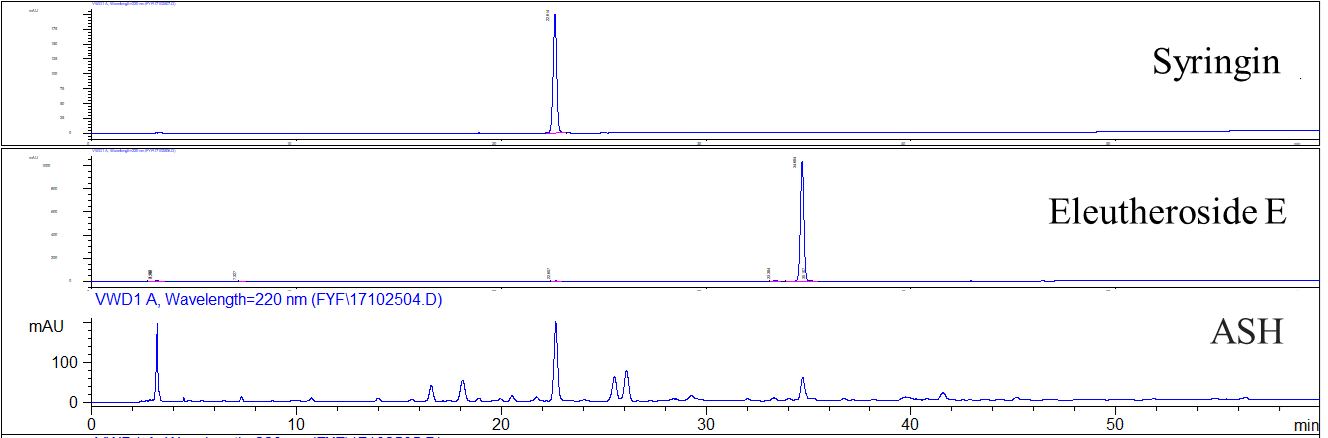


**Figure S1** HPLC diagram of syringin and eleutheroside E standard samples and ASH extracts. Syringin and eleutheroside E standard samples were dissolved in methanol and the concentration were 0.05 mg/mL and 0.5 mg/mL, respectively. *Acanthopanax senticosus* tablets were processed to extract, and volume to 25ml with methanol.

HPLC was performed on a DIKMA Platisil ODS-C18 (250×4.6 mm, 5 μm) column with a gradient mobile phase acetonitrile (A) and 0.1% aqueous phosphate (B). The flow rate was 1 mL/min, and the linear gradients were as follows: 95% B at 0 min; 53% B at 60 min. Column temperature: 25°C; UV detector, detection wavelength: 220 nm; injection volume: 10 μL.


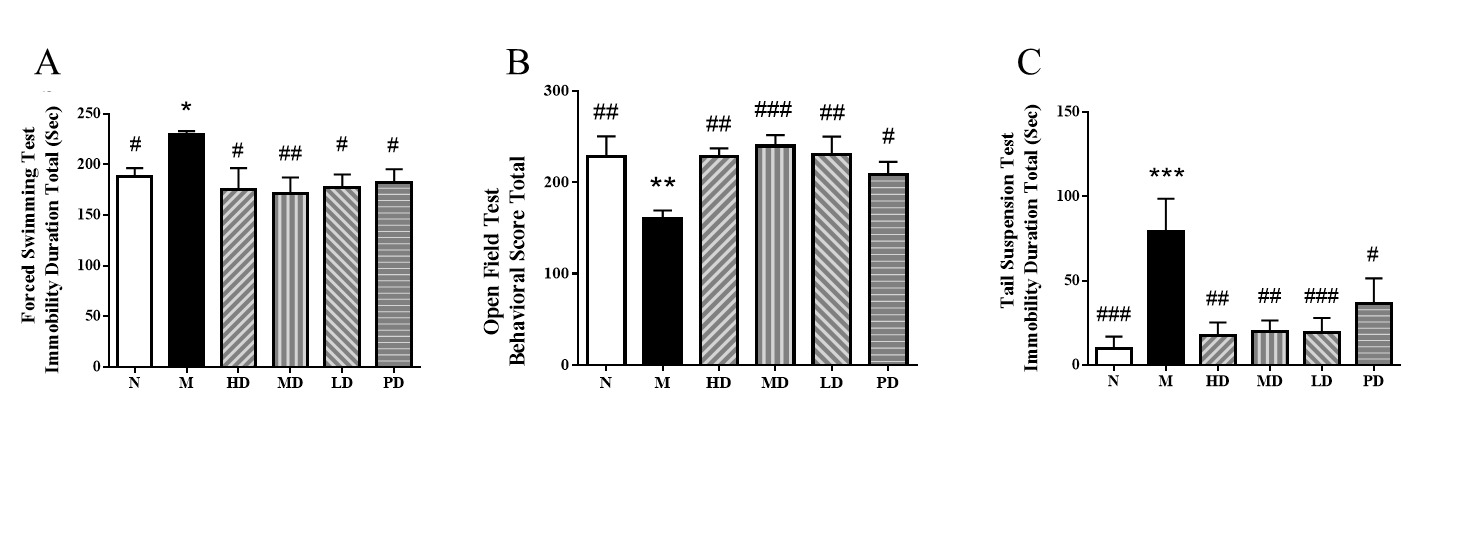


**Figure S2** Effects of ASH on depression-like behavior in CUMS model mice (n = 6). (A) Forced swimming test; (B) Open field test; (C) Tail suspension test. * P < 0.05, ** P < 0.01, *** P < 0.001, compared with N group; # P < 0.05, ## P < 0.01, ### P < 0.001, compared with M group.


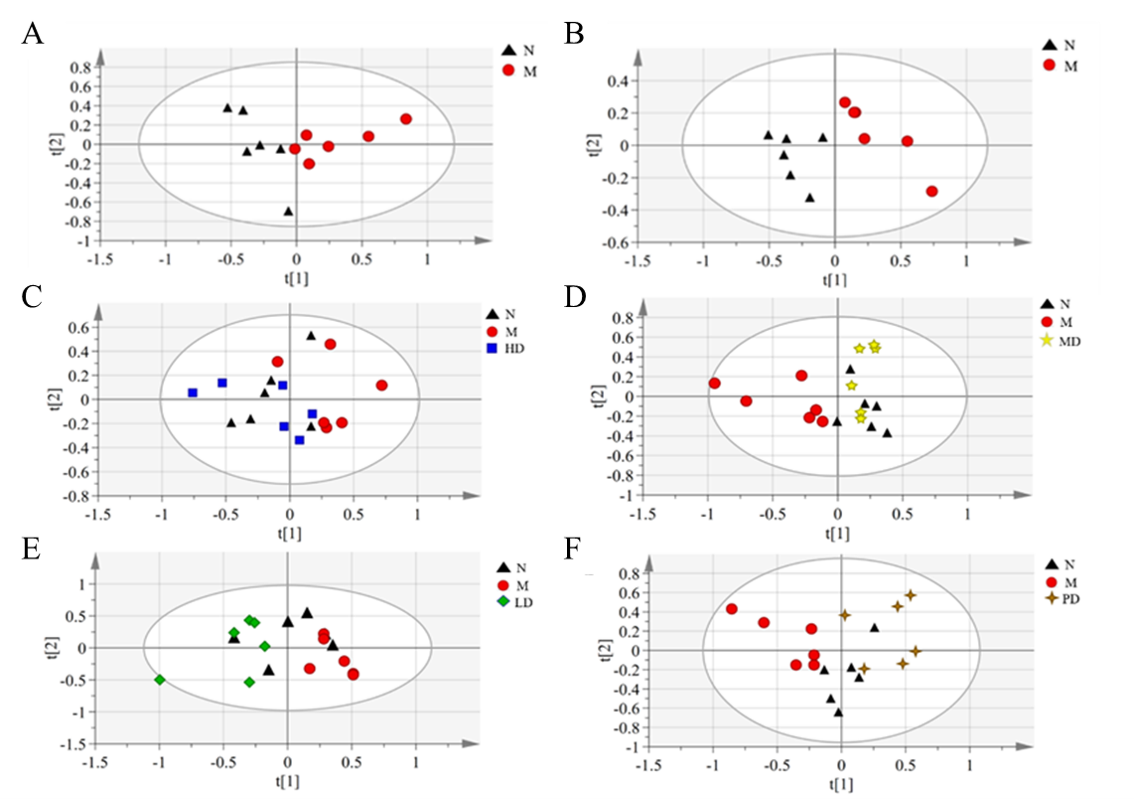


**Figure S3** Multi-dimensional statistical analysis of liver metabolism profiles of CUMS mice in each group (n = 6). (A) PCA score between N group and M group (R^2^X = 0.669, Q^2^ = 0.161); (B) PLS-DA score between N group and M group (R^2^X = 0.452, R^2^Y = 0.890, Q^2^ = 0.479); (C) PCA score among HD group, N group and M group (R^2^X = 0.617, Q^2^ = 0.209); (D) PCA score among MD group, N group and M group (R^2^X = 0.711, Q^2^ = 0.100); (E) PCA score among LD group, N group and M group (R^2^X = 0.664, Q^2^ = 0.236); (F) PCA score among PD group, N group and M group (R^2^X = 0.735, Q^2^ = 0.239).

**
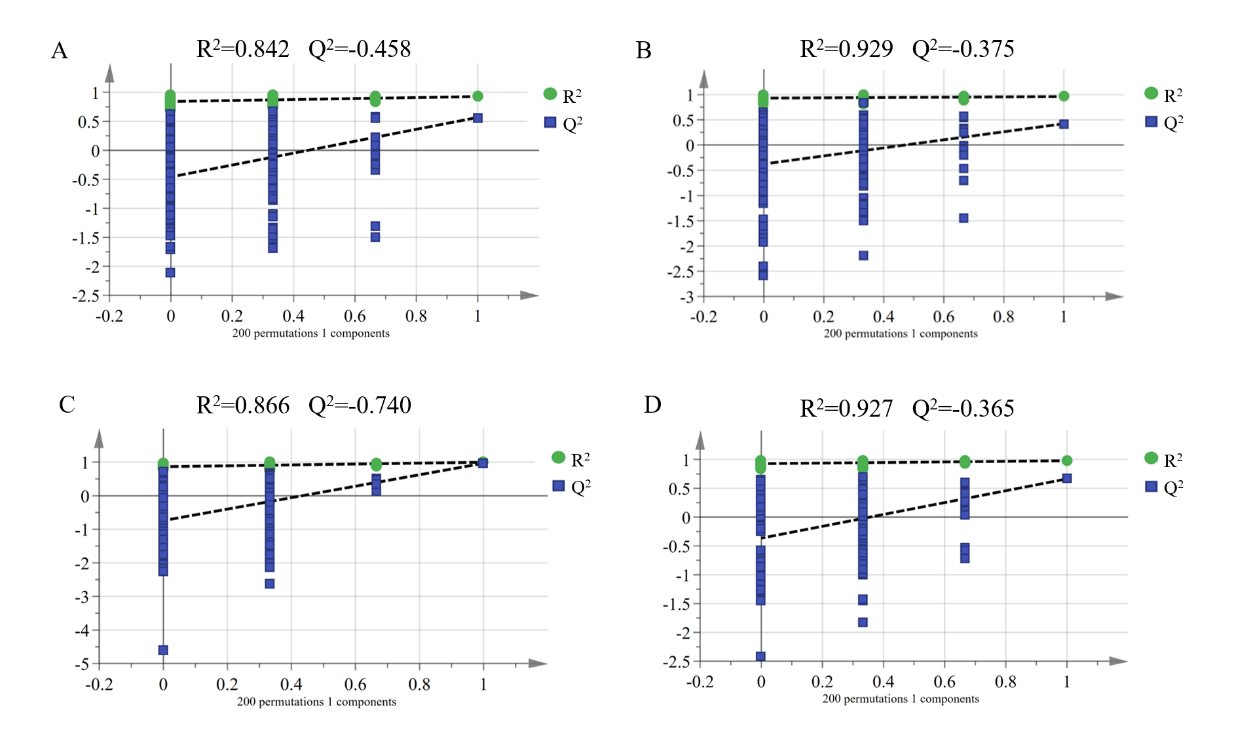
Figure S4** Permutation test of OPLS-DA score. (A) HD group vs. M group; (B) MD group vs. M group; (C) LD group vs. M group; (D) PD group vs. M group.


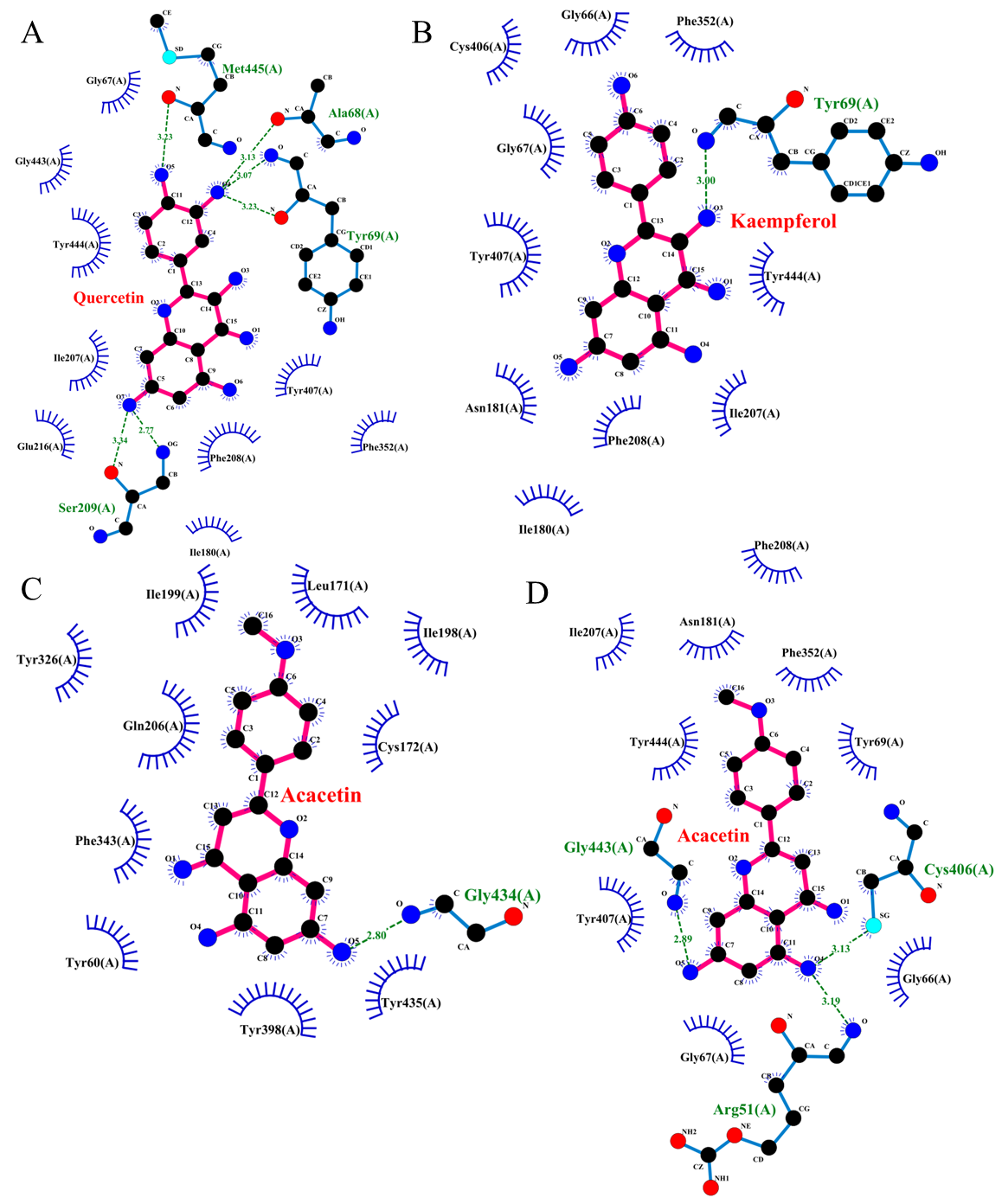


**Figure S5** Molecular docking charts of quercetin (MAOA (A)), kaempferol (MAOA (B)) and acacetin (MAOA (C) and MAOB (D)).
